# The BAP31/miR-181a-5p/RECK axis promotes angiogenesis in colorectal cancer *via* fibroblast activation

**DOI:** 10.3389/fonc.2023.1056903

**Published:** 2023-02-21

**Authors:** Qi Zhang, Changli Wang, Ruijia Li, Jingjing Liu, Jiyu Wang, Tianyi Wang, Bing Wang

**Affiliations:** College of Life Science and Health, Northeastern University, Shenyang, Liaoning, China

**Keywords:** BAP31, exosomes, fibroblast activation, angiogenesis, miR-181a-5p

## Abstract

**Background:**

B-cell receptor–associated protein 31 (BAP31) has been recognized as a tumor-associated protein and has largely been shown to promote metastasis in a variety of cancers. Cancer metastasis arises through multistep pathways, and the induction of angiogenesis is shown to be a rate-limiting step in the process of tumor metastasis.

**Methods and results:**

This study explored the effect of BAP31 on colorectal cancer (CRC) angiogenesis by regulating the tumor microenvironment. First, exosomes from BAP31-regulated CRCs affected the transition of normal fibroblasts to proangiogenic cancer-associated fibroblasts (CAFs) in vivo and in vitro. Next, microRNA sequencing was performed to analyze the microRNA expression profile of exosomes secreted from BAP31- overexpressing CRCs. The results indicated that the expression of BAP31 in CRCs significantly altered the levels of exosomal microRNAs, such as miR-181a- 5p. Meanwhile, an in vitro tube formation assay showed that fibroblasts with high levels of miR-181a-5p significantly promoted endothelial cell angiogenesis. Critically, we first identified that miR-181a-5p directly targeted the 3'-untranslated region (3′UTR) of reversion-inducing cysteine-rich protein with kazal motifs (RECK) using the dual-luciferase activity assay, which drove fibroblast transformation into proangiogenic CAFs by upregulating matrix metalloproteinase-9 (MMP-9) and phosphorylation of mothers against decapentaplegic homolog 2/Mothers against decapentaplegic homolog 3 (Smad2/3).

**Conclusion:**

Exosomes from BAP31-overexpressing/BAP31-knockdown CRCs are found to manipulate the transition of fibroblasts into proangiogenic CAFs by the miR-181a-5p/RECK axis.

## 1 Introduction

Colorectal cancer (CRC) morbidity and mortality have been mitigated recently through appropriate screening and surveillance (1), but CRC remains one of the most lethal cancers (2, 3). Angiogenesis is a cancer hallmark that obtains oxygen and other nutrients (4) by activating new capillary formation from the surrounding tissues into the tumor (5, 6).Tumor growth has been reported to be stagnant within a diameter of 1∼2 mm without blood vessels (7). In translational medicine, antiangiogenic drugs, such as bevacizumab, axitinib, sorafenib, and sunitinib, have already been approved by the Food and Drug Administration (FDA) and have shown clinical efficacies in multiple carcinoma patients (8). Accordingly, targeting angiogenesis is a successful strategy for treating cancer.

Recently, the role of the tumor microenvironment (TME) has been extensively investigated, and diverse cell populations in the TME communicate to control tumor progression through cytokines and other substances in autocrine and paracrine manners (9). Among the diverse cell populations of the TME, fibroblasts, as a key component, can be transformed into cancer-associated fibroblasts (CAFs), which lead to tumor neovascularization by secreting of angiogenic factors. Exosomes, ranging from 50 to 150 nm in size, are the major components of extracellular vesicles (EVs) (10). As naturally occurring endogenous carriers in the TME, exosomes shuttle signaling molecules between nearby and remote cells by transmitting intracellular functional biomacromolecules, such as proteins, DNA, and coding and noncoding RNAs (11–13). MicroRNAs (miRNAs), as small noncoding RNAs, regulate the production of more than one-third of human mRNAs by posttranscriptional silencing of gene expression (14, 15). Emerging evidence supports the notion that miRNAs modulate numerous hallmarks of cancer, including proliferation, apoptosis, metastasis, and angiogenesis (16, 17).

Advanced-stage tumors with gene mutations portend a worse prognosis largely because of their more aggressive biology (3). B-cell receptor–associated protein 31 (BAP31), a multifunctional integral protein of the endoplasmic reticulum (ER) membrane, has been reported in our previous studies to regulate the fate of multiple molecules and participate in multiple cellular processes in tumor and disease development (18, 19), neuroinflammation (20), and immunomodulation (21). Recently, BAP31 was discovered to function as a newly defined cancer/testis antigen (CTA) (22) and confirmed to be associated with progression and metastasis in cervical cancer (23). As reported in CRC, the expression of BAP31 was correlated with advanced clinical stage and had increased in clinical stage II and III cases most obviously (24). In addition, BAP31 was highly expressed in liver metastatic CRC tissues compared with primary CRC tissues (25). BAP31 has been further reported to regulate migration and invasion in ovarian (26), cervical (22), and lung cancers (27). Cancer metastasis arises through multistep pathways, and the induction of angiogenesis is shown to be a rate-limiting step in the process of tumor metastasis (28, 29). However, little is known about whether the aberrant expression of BAP31 in CRC affects the biological functions of surrounding tissues in the TME.

In this study, we investigated the role of exosomes from CRCs with the aberrant expression of BAP31 in the fibroblast transition. By systematically analyzing the miRNA expression profiles of exosomes from BAP31-overexpressing CRCs, we studied the effect of the expression of BAP31 on the levels of exosomal miRNAs in CRCs. Furthermore, we demonstrated that the effect of BAP31 expression on angiogenesis in the tumor microenvironment by the BAP31/miR-181a-5p/RECK axis.

## 2 Materials and methods

### 2.1 Cell lines

Human umbilical vein endothelial cells (HUVECs), human normal lung fibroblast cells (WI-38), human CRCs (HCT116 and DLD-1), mouse embryonic fibroblasts (NIH/3T3), and mouse CRCs (MC38) were cultured in the Dulbecco’s modified Eagle’s medium (DMEM), supplementing with 10% fetal bovine serum, streptomycin (100 U/ml), and penicillin (100 U/ml). They were cultured in a humidified atmosphere of 5% CO_2_ at 37°C.

BAP31-overexpressing CRCs and BAP31-knockdown CRCs were established as previously (18). All of the constructed plasmids were verified by sequencing. Real-time Quantitative Polymerase Chain Reaction (qPCR) and Western blotting analysis were used to detect gene expressions.

### 2.2 Exosomes isolation

Exosome isolation, purification, and storage were performed as reported (30). The pellets containing exosomes were collected by high-speed ultracentrifugation and resuspended in phosphate-buffered saline carefully. The amount of exosomes was measured by a BCA Protein Assay kit (KeyGEN BioTECH).

### 2.3 Nanoparticle tracking

The NanoSight NS300 equipped with sCMOS camera was used for real-time characterization of the exosomes. Measurement for nanoparticle tracking analysis was performed by a NanoSight LM10 system (NanoSight, Amesbury, United Kingdom). The capture settings and analysis settings were performed manually according to the manufacturer’s instructions.

### 2.4 Confocal laser scanning

Exosomes were fluorescently labeled using PKH67 membrane dye (Sigma). Then, the exosomes were cocultured with fibroblasts for 24 h. Nuclei were stained with Hoechst33342 for seconds, and the coverslips were sealed with resistant fluorescence quenching liquid. The fluorescence signals were analyzed by Leica SP5 confocal laser scanning microscope (Leica Microsystems, Wetzlar, Germany).

### 2.5 Immunohistochemistry

Tumor tissues were fixed by 4% paraformaldehyde, paraffin-embedded and sectioned at 5 μm. Then, tissue sections were deparaffinized in xylene and rehydrated in a series of graded alcohols. The tissue sections were stained with hematoxylin and eosin or immunohistochemically stained with antibodies following the manufacturer’s instructions. The staining was observed and photographed with a microscope (Olympus, Model BX40F4, Tokyo, Japan). All antibodies used in this study are shown in **Table S1**.

### 2.6 Western blotting

The protein samples were separated on 6%–15% Sodium Dodecyl Sulfate Polyacrylamide Gel Electrophoresis (SDS-PAGE) and transferred to Polyvinylidene Fluoride (PVDF) membranes. The membranes were blocked by 5% bovine serum albumin and incubated with specific primary antibodies overnight at 4°C and incubated with the appropriate secondary antibodies. Finally, the blots were developed using the Enhanced Chemiluminescence Kit. The ratios of target protein band intensities relative to that of housekeeping protein using ImageJ software, which reflect the change of expression level, were calculated. All antibodies used in this study are shown in **Table S1**.

### 2.7 Real-time polymerase chain reaction

Total RNA was extracted using Invitrogen TRIzol Reagent. For miRNA quantification, 100 ng of total RNA was directly reverse-transcribed using stem-loop primers. For mRNA analyses, cDNA was synthesized from 2 μg of total RNA, using oligo (dT) primers. Quantitative real-time PCR was performed using the SYBR Green PCR Master Mix in a final volume of 20 μl on the Bio-Rad CFX96TM Real-Time System. The expressions of miRNAs or mRNAs were normalized to U6 or Glyceraldehyde-3-phosphate dehydrogenase (GAPDH), respectively. Results were presented as the relative quantification based on the calculation of 2^−ΔΔCt^. All primers used in this study are shown in **Table S2**.

### 2.8 Cell viability assay

Cells were seeded in 96-well plates, and the plate was incubated at 37°C in a 5% CO_2_ incubator. After treatment, 3-(4, 5)-dimethylthiahiazo (-z-y1)-3, 5-di-phenytetrazoliumromide (MTT) (final concentration: 5 mg/ml) was added to each well, and the plate was incubated for 4 h. Before measuring, the supernatant was removed, and 150 μl of dimethyl sulfoxide was added to solubilize the formazan crystals. The optical density of the samples was measured by a Synergy H1 microplate reader (Biotek, USA) at a wavelength of 490 nm.

### 2.9 Transwell assay

The Boyden chamber cell assay was used to assess cell migration longitudinally. The treated cells were seeded onto the top chamber inserts with 8-μm pores (Corning, NY). After incubation, the migrating cells in the bottom chamber were fixed by methanol and then stained with 0.1% crystal violet. The migrating cells were observed from three randomly chosen fields and quantified by manual counting.

### 2.10 Wound healing assay

The cells were seeded in 24-well plates. Then, the attached cells in the plate were scraped away using a pipette tip. After phosphate-buffered saline cleaning, the scratched monolayers were enriched with condition medium. Images were taken using an inverted fluorescence microscope after incubation for 24h or 48h. The migrating cells were observed from three randomly chosen fields and quantified by manual counting.

### 2.11 Tube formation assay

Matrigel matrix (Corning) was plated in 96-well plates and incubated at 37°C to polymerize the Matrigel. The HUVECs were seeded on the Matrigel-coated well in a final volume of 100 μl. The morphogenesis of endothelial cells was recorded using an inverted fluorescence microscope. Results were quantified by measuring tube lengths and areas using ImageJ software.

### 2.12 Dual-luciferase activity assay

The sequences of wild-type (WT) or mutated RECK 3′UTR were inserted into psiCHECK-2 vector; then, the constructed plasmids and miRNA mimics were cotransfected into cells. Firefly and Renilla luciferase activities were determined using the Dual-Luciferase Reporter Gene Assay Kit (Beyotime). The fluorescence intensity of the samples was measured by a Synergy H1 microplate reader (Biotek, USA). Relative luciferase activity was calculated by normalizing Renilla/Firefly values.

### 2.13 Animal experiments

Female BALB/C mice (6–8 weeks) were purchased from Changsheng biotechnology, and they were fed in an SPF (specific pathogen–free) animal facility. CRCs and fibroblasts were separately suspended at 2 × 10^6^/ml in DMEM-containing antibiotics and inoculated subcutaneously into the back of these mice that were maintained in the SPF environment (0.1 ml per site/animal). The animal study was reviewed and approved by Animal Care and Use Committee of the Northeastern University Committee, China.

### 2.14 Statistical analysis

All the experiments were performed at least in triplicate and in independent biological replicates. Statistical analyses were performed using Prism 8.0 software (GraphPad, La Jolla, CA, USA). The significance of mean values between two groups was analyzed by Student’s t-test. Statistically significant changes are indicated (*p < 0.05, **p < 0.01, and ***p < 0.001).

## 3 Results

### 3.1 Exosomes from CRCs were uptakeninto fibroblasts

**Figure 1:**
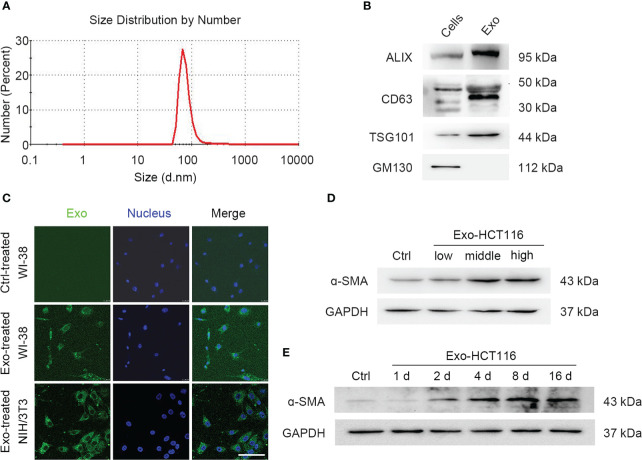
Exosomes from CRCs were uptaken into fibroblasts. **(A)** Zetasizer nanoparticle size analysis shows the particle size from HCT116 cells. **(B)** Western blot analysis shows exosomal biomarkers. Exosomal specific markers: TSG101, CD63, and Alix; Cell-specific marker: GM130. **(C)** Confocal microscope images show fibroblast uptake of PKH67-labeled exosomes for 24 h. Scale bar, 100 μm. **(D)** Western blot analysis shows the α-SMA expression under the treatment of exosomes of series concentrations (low, middle, and high concentration represent 0.3, 3, and 30 μg/ml, separately). **(E)** Western blot analysis shows the α-SMA expression under the treatment of exosomes during a period time.

Tumors have been reported to present a particular microenvironment comprising multiple cell populations, leading to the acquisition of malignant features and promoting tumor progression (31, 32). In the TME, quiescent fibroblasts can be reprogrammed into proangiogenic CAFs (33, 34). Considering the role of exosomes in cell communication, we first identified exosomes secreted by CRCs. The particles collected by ultracentrifugation from the culture supernatant of CRCs were characterized. Nanoparticle tracking analysis showed that the size of the particles ranged from 50 to 150 nm (**Figure 1A**). In addition, exosome marker proteins were enriched in isolated particles, whereas golgiosome marker proteins were only present in cells (**Figure 1B**). The above results confirmed that the isolated particles were exosomes.

Furthermore, we employed a coculture system to investigate whether the exosomes secreted by CRCs can be absorbed by fibroblasts. After coculturing for 24 h, PKH67-labeled exosomes were observed to accumulate in the cytoplasm of both human fetal lung fibroblasts WI-38 and murine fibroblasts NIH/3T3 (**Figure 1C**). Western blotting was used to analyze the expression of α–smooth muscle actin (α-SMA), which serves as a marker for the differentiation of fibroblasts into CAFs (**Figures 1D, E**; **Figures S1A, B**). Similar results were obtained for DLD-1–derived exosomes (**Figures S1C, D**). These results indicated that CRC-derived exosomes are involved in the communication between CRCs and fibroblasts.

### 3.2 Exosomes from BAP31-overexpressing CRCs promoted fibroblast activation

**Figure 2:**
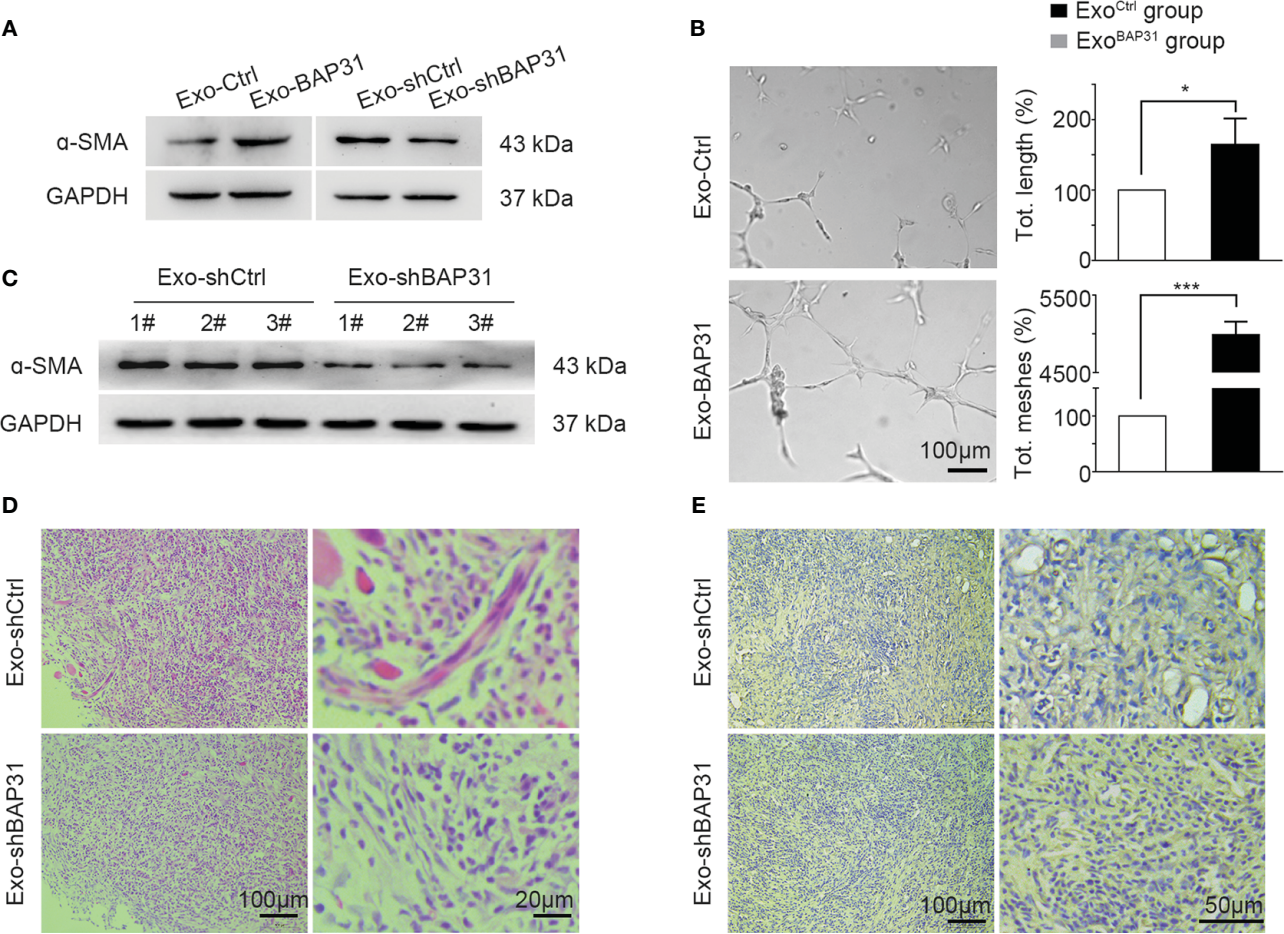
Exosomes from BAP31-overexpressing CRCs promoted fibroblast activation. Fibroblasts were treated by exosomes from BAP31-overexpressing/BAP31-knockdown CRCs. **(A)** Western blot analysis shows the α-SMA expression in fibroblasts. **(B)** The endothelial cells were treated by the conditioned medium from the fibroblasts. Tube formation analysis shows the angiogenic ability of the endothelial cells. Scale bar, 100 μm. Mean ± SEM are provided (n = 3). *p < 0.05 and ***p < 0.001. Mouse fibroblasts (NIH/3T3) were treated by exosomes from BAP31-knockdown CRCs. Mouse CRCs (MC38) were mixed with the fibroblasts and then injected subcutaneously into BALB/C mice. **(C)** Western blot analysis shows the α-SMA expression in tumor tissues. **(D)** H&E staining images show fibroblast differentiation in tumor tissues. **(E)** Immunohistochemistry (IHC) staining (CD31) images show the vascular structures in tumor tissues.

We focused on exploring whether exosomes from BAP31-overexpressing/BAP31-knockdown CRCs promote the transition of fibroblasts into proangiogenic CAFs. BAP31 was overexpressed or knocked down by transfection of pcDNA3.1(−)-BAP31-Flag or lentivirus packed plko.1-puro-shBAP31 in CRCs to obtain stable cell lines (**Figures S2A, B**). Exosomes derived from BAP31-overexpressing (Exo-BAP31) or BAP31-knockdown (Exo-shBAP31) CRCs were analyzed to determine the correlation of BAP31 with fibroblast activation through exosomes. The results revealed that exosomes from BAP31-overexpressing CRCs upregulated the expression of α-SMA in fibroblasts (**Figure 2A**; **Figures S2C, D**). *In vitro* tube formation assays further supported the hypothesis that exosomes from BAP31-overexpressing CRCs increase the proangiogenic effect of fibroblasts (**Figure 2B**).

Furthermore, exosomes from BAP31-knockdown CRCs were cocultured with fibroblasts for 4 days, and then, the fibroblasts mixed with mouse CRCs (MC38) were injected subcutaneously into BALB/C mice. The expression of α-SMA was suppressed in the Exo-shBAP31 group compared with that in the Exo-Ctrl group (**Figure 2C**). The Hematoxylin and Eosin stain (H&E) staining results showed that Exo-shBAP31 markedly decreased myofibroblasts, which exhibited blue fluorescence because of abundant rough ER; nevertheless, CAFs, which showed purple cytoplasm with fibrillary quality and typically active nuclei, significantly increased (**Figure 2D**). In addition, less plentiful microvessel density was observed in the Exo-shBAP31 group (**Figure 2E**). Meanwhile, we found that the growth of tumors was significantly decreased in the Exo-shBAP31 group mice compared with that in the Exo-shCtrl group mice (**Figures S2E, F**), which was primarily due to less plentiful microvessels to restrict the growth of tumors.

### 3.3 Exosomal miR-181a-5p was positively regulated by the expression of BAP31

**Figure 3:**
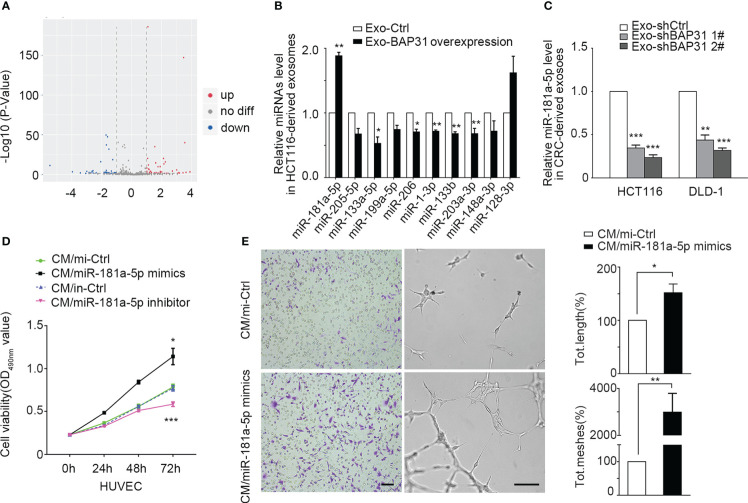
Exosomal miR-181a-5p was positively regulated by the expression of BAP31. **(A)** Heatmap of microarray data shows the differences of exosomal miRNAs from BAP31-overexpressing CRCs. **(B)** qPCR analysis shows the expression of exosomal miRNAs from BAP31-overexpressing CRCs. Mean  ± SEM are provided (n = 3). *p < 0.05 and **p < 0.01. **(C)** qPCR analysis shows the expression of exosomal miR-181a-5p from BAP31-knockdown CRCs. Mean  ± SEM are provided (n = 3). ***p < 0.001. Fibroblasts were transfected with miR-181a-5p mimics to increase the miR-181a-5p expression of fibroblasts. The endothelial cells were treated by the conditioned medium from the fibroblasts. **(D)** MMT assay shows the endothelial cells proliferation. Mean  ± SEM are provided (n = 3). *p < 0.05 and ***p < 0.001. **(E)** Transwell and tube formation assays show the endothelial cells migration and angiogenesis. Scale bar, 100 μm. Mean  ± SEM are provided (n = 3). *p < 0.05 and **p < 0.01.

miRNA sequencing was performed to analyze the effect of BAP31 on the miRNA expression profile in CRC-derived exosomes. The miRNA sequencing data were provided by Lianchuan Biotechnology using ACGT101-miR software (LC Sciences, Houston, Texas, USA). A total of 152 mature miRNAs in exosomes from BAP31-overexpressing CRCs were obtained (**Figure 3A**). Furthermore, 10 miRNAs were selected for further investigation due to their presence with ≥ 100 reads in both test groups and |log_2_ (fold change)| ≥ 0.35 (**Figure S3A**). To identify the miRNAs affected by the expression of BAP31, we first verified the 10 differential expressed miRNAs in exosomes from BAP31-overexpressing HCT116 by qPCR (**Figures 3B**, **S3B**). Meanwhile, we further identified these exosomal miRNAs from BAP31-knockdown CRCs (HCT116-shBAP31 and DLD-1–shBAP31) by qPCR (**Figures 3C**; **S3C, D**). The results revealed that miR-181a-5p was significantly affected in both BAP31-overexpressing and BAP31-knockdown cell lines. These results suggested that miR-181a-5p expression in exosomes is positively correlated with the expression of BAP31 in CRCs.

Intriguingly, miR-181a-5p has previously been reported to affect tumor angiogenesis (35, 36), but the role is still controversial. To further investigate the angiogenic functions of miR-181a-5p on the transition of fibroblasts into proangiogenic CAFs, miR-181a-5p mimics and an inhibitor were separately transfected into fibroblasts. The Condition Medium (CM) from the mimic group was collected and cocultured with HUVECs to observe whether miR-181a-5p promotes fibroblast transition into proangiogenic CAFs. Treatment with miR-181a-5p mimics significantly promoted the proliferation, migration, and angiogenesis of HUVECs (**Figures 3D, E**; **Figures S3E, F**). These results indicated that miR-181a-5p enhances the transition of fibroblasts into proangiogenic CAFs.

### 3.4 The 3′UTR of RECK was a functional target of miR-181a-5p

**Figure 4:**
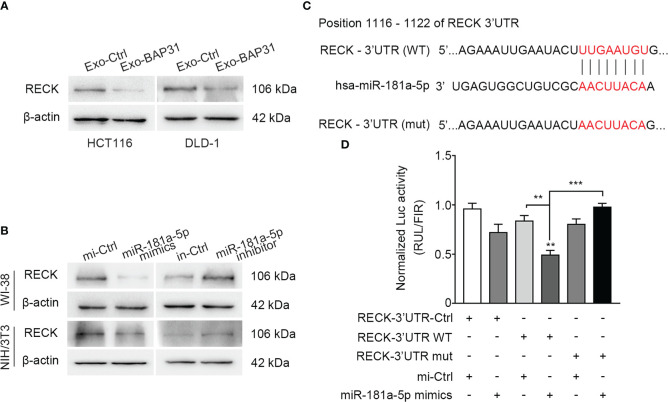
The 3′UTR of RECK was a functional target of miR-181a-5p. **(A)** Fibroblasts were treated by exosomes from BAP31-overexpressing CRCs. Western blot analysis shows the RECK expression in the fibroblasts. **(B)** Fibroblasts were transfected with miR-181a-5p mimics to increase the miR-181a-5p expression of fibroblasts. Western blot analysis shows the RECK expression in the fibroblasts. **(C)** Schematic diagram shows the wild-type (WT) sequences of RECK 3′-UTR binding sites matching for miR-181a-5p, and the mutant (mut) sequences of RECK 3′-UTR. **(D)** Luciferase activities reporter shows the binding effect of miR-181a-5p to the RECK 3′-UTR in HEK-293T. Mean ± SEM are provided (n = 3). **p < 0.01 and ***p < 0.001.

miRNA has been reported to exert its biological effect *via* efficient silencing of mRNAs to reprogram the target cell transcriptome (37). To explore the mechanism by which miR-181a-5p reprograms fibroblasts and to predict its potential downstream targets, miRNA target-predicting algorithms (TargetScan, miRDB, and miRanda) were utilized. Among the seven overlapping potential targets in all databases (**Table S4**), further analysis confirmed that RECK was significantly changed at the protein level in fibroblasts cocultured with exosomes from BAP31-overexpressing CRCs (**Figures 4A**; **Figures S4A, B**); however, the other genes had little significant differences (data not shown). Thus, RECK was hypothesized to be a target gene of miR-181a-5p.

Next, to identify RECK as a direct target of miR-181a-5p in fibroblasts, we assessed the protein level of RECK in fibroblasts transfected with miR-181a-5p mimics or inhibitors. Consistent with the results from the abovementioned prediction assay, miR-181a-5p markedly decreased the expression level of RECK but had little effect at the mRNA level (**Figure 4B**; **Figures S4C–H**). Collectively, these results indicated that miR-181a-5p exerts its inhibitory effect on RECK through posttranscriptional silencing. According to the binding sites of miR-181a-5p targeting the RECK 3′UTR (**Figure 4C**), a dual-luciferase reporter gene was constructed by inserting the RECK 3′UTR WT binding sequence of miR-181a-5p into the 3′ end of Renilla luciferase in the psiCHECK-2 vector. As a mutation control (mut), the predicted miR-181a-5p response element (8 nt) was converted to the complementary sequence to eliminate potential miR-181a-5p binding. The results showed that the Renilla luciferase activities were inhibited in the WT group because of its direct binding to miR-181a-5p mimics (**Figure 4D**). In summary, these data provided clear evidence of the biologically effective interaction of miR-181a-5p and the 3′UTR of RECK.

### 3.5 BAP31 promoted fibroblast activation *via* exosomal miR-181a-5p

**Figure 5:**
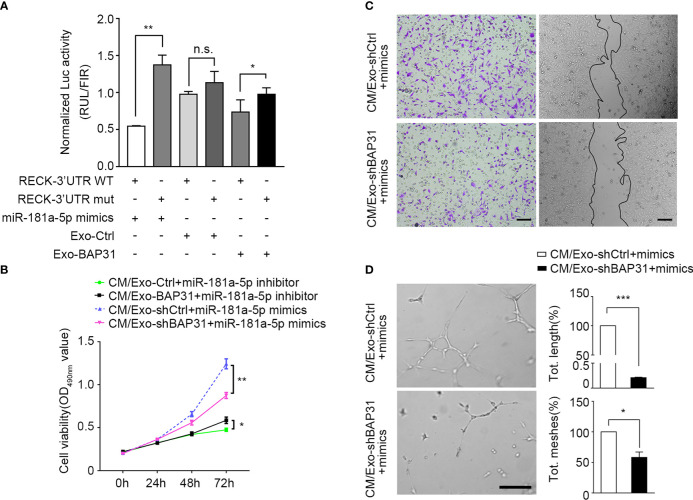
BAP31 promoted fibroblast activation *via* exosomal miR-181a-5p. Fibroblasts were transfected with miR-181a-5p mimics or treated by exosomes from CRCs. **(A)** Luciferase activities reporter shows the binding effect of miR-181a-5p and BAP31-induced CRC exosomes to the RECK 3′-UTR. Mean ± SEM are provided (n = 3). *p < 0.05 and **p < 0.01; n.s., non-significance. The endothelial cells were treated by the conditioned medium from the fibroblasts. **(B)** MMT assay shows the endothelial cells proliferation. Mean ± SEM are provided (n = 3). *p < 0.05 and **p < 0.01. **(C)** Transwell and wound healing assays show the endothelial cells migration. Scale bar, 100 μm. **(D)** Tube formation assay shows the endothelial cells angiogenesis. Scale bar, 100 μm. Mean  ± SEM are provided (n = 3). *p < 0.05 and ***p < 0.001.

To explore whether exosomes from BAP31-overexpressing/BAP31-knockdown CRCs alter fibroblast transition by miR-181a-5p, HEK-293T cells were transfected with the constructed dual-luciferase reporter vector psiCHECK-2-RECK-3′UTR-WT (WT) or psiCHECK-2-RECK-3′UTR-mut (mut). The results showed that the Renilla luciferase activities significantly decreased in cells treated with exosomes from BAP31-overexpressing CRCs, which is consistent with the results of the miR-181a-5p mimic group (**Figure 5A**). Consequently, the results demonstrated that exosomes from BAP31-overexpressing CRCs target the binding sites of miR-181a-5p on the RECK 3′UTR.

To investigate whether fibroblasts activated through the BAP31/miR-181a-5p axis gain proangiogenic CAF-like features, CM from fibroblasts was collected and cocultured with HUVECs under the stimulus of miR-181a-5p mimics or exosomes from BAP31-knockdown CRCs. As shown in **Figure 5B**, exosomes from BAP31-knockdown CRCs abated the pro-proliferation effect of fibroblast transfection with miR-181a-5p mimics. In addition, the miR-181a-5p inhibitor markedly inhibited the proliferation of HUVECs and was neutralized by exosomes from BAP31-overexpressing CRCs. The biological correlation and proangiogenic function of the BAP31/miR-181a-5p axis were further identified *in vitro* by wound healing assay/Transwell and tube formation assays (**Figures 5C, D**). These results provide evidence that BAP31 activates fibroblasts to promote angiogenesis through exosomal miR-181a-5p in CRCs.

### 3.6 miR-181a-5p activated fibroblasts by upregulating MMP-9 and phosphorylation of Smad2/3

**Figure 6:**
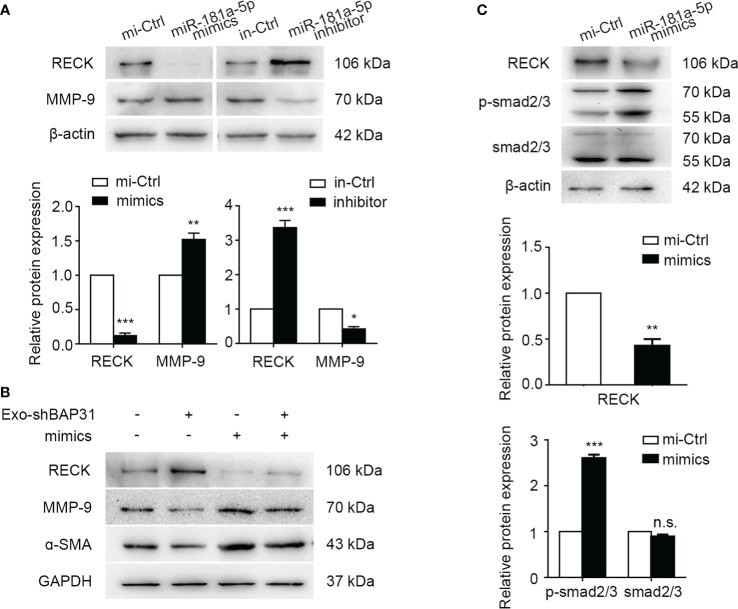
miR-181a-5p activated fibroblasts by upregulating MMP-9 and phosphorylation of smad2/3. **(A)** Western blot analysis shows the expression of RECK and MMP-9 in fibroblasts transfected with miR-181a-5p mimics/inhibitor. Mean ± SEM are provided (n = 3). *p < 0.05, **p < 0.01, and ***p < 0.001. Fibroblasts were transfected with miR-181a-5p mimics or treated by exosomes from BAP31-knockdown CRCs. **(B)** Western blot analysis shows the expression of RECK, MMP-9, and α-SMA in the fibroblasts. **(C)** Western blot analysis shows the expression of the phosphorylation of Smad2/3 in fibroblasts. Mean  ± SEM are provided (n = 3). **p < 0.01 and *** *p* < 0.001; n.s., non-significance.

The RECK gene has been reported to encode a membrane-anchored glycoprotein that can negatively regulate MMP-2 and MMP-9 activities and is strongly associated with tumor angiogenesis and metastasis in various human cancers (38, 39). To explore the mechanism by which miR-181a-5p activates fibroblasts, we first analyzed the expression of MMP-2 and MMP-9 in fibroblasts following transfection with miR-181a-5p mimics/inhibitor. The results showed that exogenous miR-181a-5p increased the expression of MMP-9 but had little effect on the expression of MMP-2 in fibroblasts (**Figure 6A**, **Figures S5A, B**). Furthermore, exosomes from BAP31-knockdown CRCs were found to inhibit the expression of MMP-9 and α-SMA in fibroblasts transfected with miR-181a-5p mimics (**Figure 6B**, **Figures S5C–E**). This suggests that exosomes from BAP31-knockdown CRCs abated the effect of MMP-9 expression and fibroblast activation by miR-181a-5p mimics. Furthermore, we found that the level of MMP-9 mRNA significantly increased in fibroblasts transfected with miR-181a-5p mimics (**Figure S5F**).

Many research efforts have focused on the functions of TGF-β/Smad signaling in fibroblasts and myofibroblasts and the association of aberrant TGF-β/Smad signaling with the myofibroblast phenotype (40, 41). The upstream region of the α-SMA promoter was reported to bind to the Smad3-binding element to regulate the transcriptional activation of the α-SMA gene (42). MMP-9 has been discovered to be recruited to the fibroblast cell surface, where it activates latent Transforming growth factor beta (TGF-β) and induces myofibroblast differentiation (43). Accordingly, we investigated whether miR-181a-5p regulated the TGF-β/Smad signaling pathway. The results demonstrated that miR-181a-5p significantly enhances the phosphorylation of Smad2/3 in fibroblasts (**Figure 6C**), and miR-181a-5p has little significant effect on TGF-β (data not shown).

Together, the abovementioned results made it clear that miR-181a-5p transforms fibroblasts into CAFs by upregulating MMP-9 expression and activating the phosphorylation of Smad2/3.

## 4 Discussion

Neoplastic epithelial cells coexist in carcinomas with several distinct stromal cell types that together create the microenvironment of the cancer cells. The contribution of the stromal microenvironment to the development of a wide variety of tumors has been supported by extensive experimental models and clinical evidence (44–46). The presence and contribution of CAFs in CRC have been well documented; however, the specific mechanism of the transition of normal fibroblasts into CAFs has been largely unexplored.

Stromal regions microdissected from human cancers have shown a high frequency of genetic alterations (47, 48). These results have raised the possibility that an accumulation of genetic alterations may contribute to the generation of the tumor microenvironment, thereby enhancing malignant phenotypes. We noted that fibroblasts cocultured within exosomes from BAP31-overexpressing/BAP31-knockdown CRCs acquired significant biological differences. These fibroblasts exhibited alterations in the expression of α-SMA, the characteristics of CAFs, and the effect of tumor angiogenesis.

Accordingly, we provided clear evidence that the protein level of BAP31 in CRCs affected the function of exosomes in the transition of fibroblasts into proangiogenic CAFs. Such conversion of fibroblasts suggested that exosomes from genetic alterations are already fully able to exert tumor-enhancing effects. We demonstrated that exosomes secreted from BAP31-overexpressing CRCs promote the transition of fibroblasts into CAFs via miR-181a-5p, further enhancing angiogenesis. Because the role of miR-181a-5p in CRC has been debated (35, 36), our observation provided a new explanation in which miR-181a-5p converts normal fibroblasts into proangiogenic CAFs in the TME.

A large number of miRNAs have been reported to be involved in the regulation of posttranscriptional silencing of gene expression. In this study, we confirmed the biologically direct interaction of miR-181a-5p with the 3′UTR of RECK by a dual-luciferase assay. In addition, the RECK gene is widely expressed in various human organs, but its expression is low or undetectable in many tumor-derived cell lines. Thus, it is important in the TME, especially in stromal cells, that the expression of RECK is inhibited by miR-181a-5p. Notably, RECK has been reported to promote angiogenesis in endothelial cells. In mammalian CNS ECs, RECK genetically interacts with Gpr124 to transduce Wnt7a- and Wnt7b-specific signals to promote angiogenesis (49). Meanwhile, the RECK gene could negatively regulate MMP-2 and MMP-9 activities (38, 50) and even MMP-9 mRNA (51). Thus, we analyzed the expression of MMP-2 and MMP-9 in fibroblasts transfected with miR-181a-5p mimics/inhibitor. The results showed that exogenous miR-181a-5p insignificantly affected the expression of MMP-2 (**Figures S5A, B**). Then, we found that RECK was reported to regulate MMPs at different levels, e.g., through downregulation of MMP transcription, translation or secretion or by binding and sequestering MMPs (39, 49–52). Moreover, Takagi et al. found that RECK decreased the binding of Fra-1 and c-Jun to TRE-1 within the MMP-9 promoter region, thus decreasing the MMP-9 mRNA level (51). These studies explain that miR-181a-5p regulates the expression of MMP-9 but has little effect on the expression of MMP-2. Because RECK is a membrane protein, the biochemical mechanism by which RECK enhances MMP-9 transcription has not been further determined. Despite this, we demonstrated that MMP-9 expression was elevated at the transcriptional level. MMP-9 has been reported to be recruited to the fibroblast cell surface *via* its fibronectin type II–like motifs by Lysyl Hydroxylase 3 (LH3), where it activates latent TGF-β and induces myofibroblast differentiation as shown by increased α-SMA expression (40). Finally, a feedback cascade was identified in which the miR-181a-5p/RECK axis activates fibroblasts by upregulating MMP-9 and activating the phosphorylation of Smad2/3.

In this study, we have explained that miR-181a-5p activated fibroblasts by targeting RECK. In addition, we found that exosomal miR-181a-5p can be positively regulated by the expression of BAP31 in CRCs. Nevertheless, we hardly explain the mechanism by which BAP31 regulates exosomal miR-181a-5p. Thus, the major limitation of this study is the mechanism of exosomal miR-181a-5p production. BAP31 is an integral ER-resident membrane protein, but some mature miRNAs were found to be regulated in CRCs with the abnormal expression of BAP31. Furthermore, some miRNA precursors were found to be regulated by the expression of BAP31 (data not published). Accordingly, we speculated that the expression of BAP31 may regulate some miRNA promoters. In previous studies, Transcription factor (SOX2) knockdown decreased the levels of miR-181a-5p and miR-30e-5p in breast cancer cells (53), and STAT1 bound to specific elements within the miR-181a promoter region to inhibit miR-181a expression (54); however, a gap has been existed in the research of the correlation BAP31 and transcription factors. To our knowledge, BAP31 was reported to regulate the production of the transcription factors of the M2-type macrophages by regulating CD4+ T cells (55). Thus, the mechanism whether BAP31 regulated some transcription factors to bind to miR-181a-5p promoters is still to be further explored. On the other hand, it was reported that the motifs of the FUS RNA-binding protein (FUS) displayed specific miR-181a-5p binding sites; thus, FUS could mediate miR-181a-5p packaging into EVs in CRCs (56). Although some questions remain to be addressed, we demonstrated that exosomes from BAP31-overexpressing/BAP31-knockdown CRCs manipulate the transition of fibroblasts into CAFs through miR-181a-5p, which provides evidence for exosomal miR-181a-5p production. The study highlights the important role of exosomal miR-181a-5p in the interactions between CRCs and fibroblasts. These findings may be useful in the exploration and treatment of non-coding RNA in the future.

## 5 Conclusion

In summary, we demonstrate that exosomes from BAP31-overexpressing CRCs induce the transformation of fibroblasts into proangiogenic CAFs. Further miRNA sequencing indicates that BAP31 in CRCs significantly affects the levels of miRNAs in exosomes and that miR-181a-5p is regulated by the expression of BAP31. Meanwhile, fibroblasts with high levels of miR-181a-5p significantly promote angiogenesis. In addition, miR-181a-5p directly targets the 3′UTR of the RECK gene that drives fibroblast transformation into proangiogenic CAFs by regulating MMP-9 and the phosphorylation of Smad2/3. Collectively, the results reveal that exosomes from BAP31-overexpressing/BAP31-knockdown CRCs manipulate the transition of fibroblasts into CAFs through miR-181a-5p, which may be a promising candidate for targeting angiogenesis.

## Data availability statement

The original contributions presented in the study are included in the article/**Supplementary Material**. Further inquiries can be directed to the corresponding authors.

## Ethics statement

The animal study was reviewed and approved by Animal Care and Use Committee of the Northeastern University Committee, China.

## Author contributions

QZ, CW, RL, JL, and JW performed the experiments and provided acquisition, analysis, and interpretation of data; QZ drafted the manuscript; BW and TW directed the design of the study and analyzed and approved all of the data. All authors contributed to the article and approved the submitted version.
