# Supplementary material for: The BAP31/miR-181a-5p/RECK axis promotes angiogenesis in colorectal cancer via fibroblast activation
Source: Front Oncol. 2023 Feb 21;13:1056903. doi: 10.3389/fonc.2023.1056903 (PMC9989165; doi:10.3389/fonc.2023.1056903)
Supplement: Supplementary file 1 [file DataSheet_1.docx]

***Supplementary Figures***


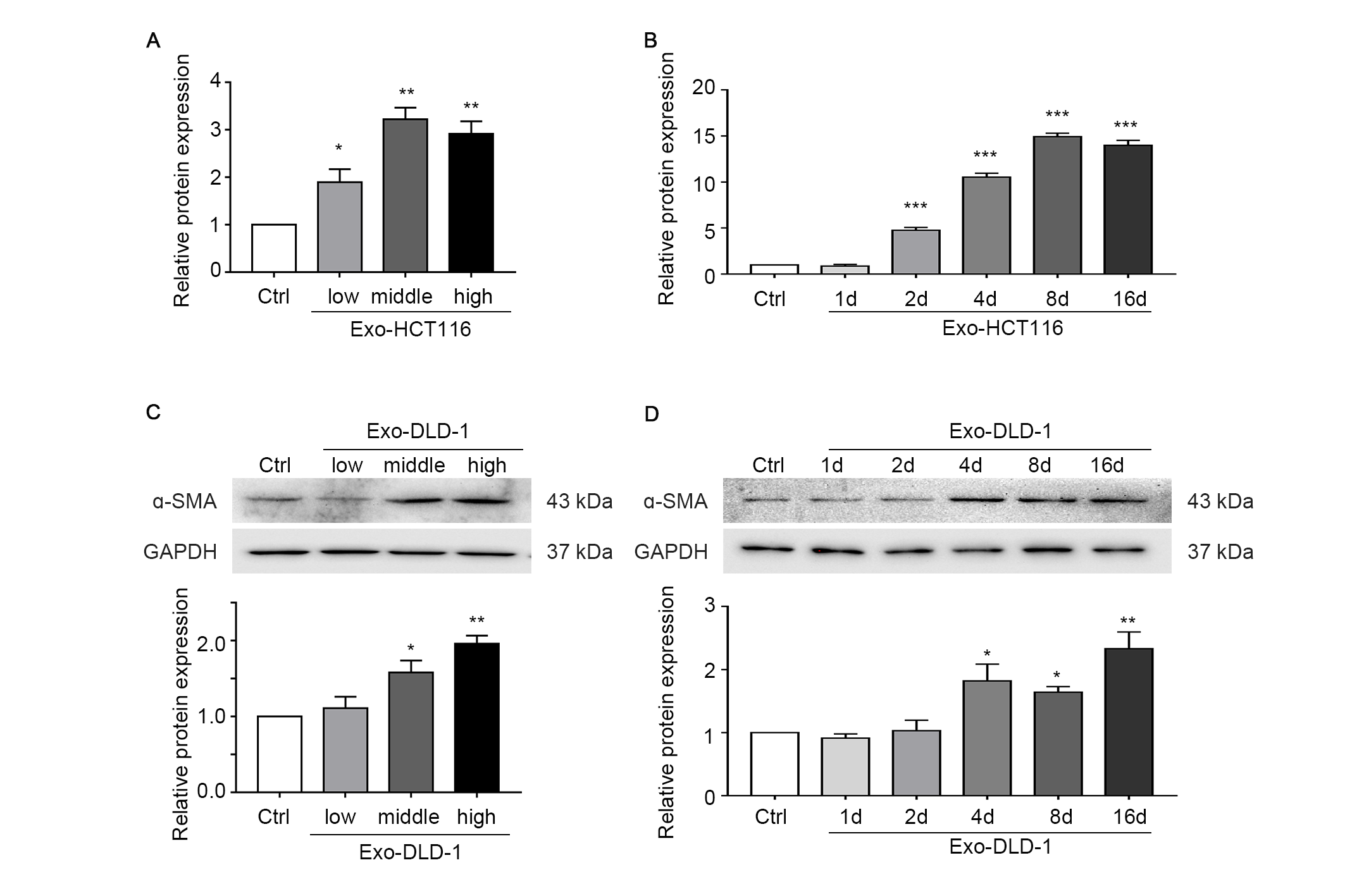
**Supplementary Figure1. Exosomes from CRCs were Uptaken into Fibroblasts.** **(A, B)** ImageJ software analyzes the relative quantification of the α-SMA expression. (low, middle and high concentration represent 0.3, 3, 30 μg/mL, separately). Mean  ± SEM are provided (n = 3). * *p* < 0.05, ** *p* < 0.01, *** *p* < 0.001. **(C)** Western blot analysis shows the α-SMA expression under the treatment of exosomes of series concentrations. Low, middle and high concentration represent 0.3, 3, 30 μg/mL, separately. Mean  ± SEM are provided (n = 3). * *p* < 0.05, ** *p* < 0.01. **(D)** Western blot analysis shows the α-SMA expression under the treatment of exosomes during a period time. Mean  ± SEM are provided (n = 3). * *p* < 0.05, ** *p* < 0.01.

**
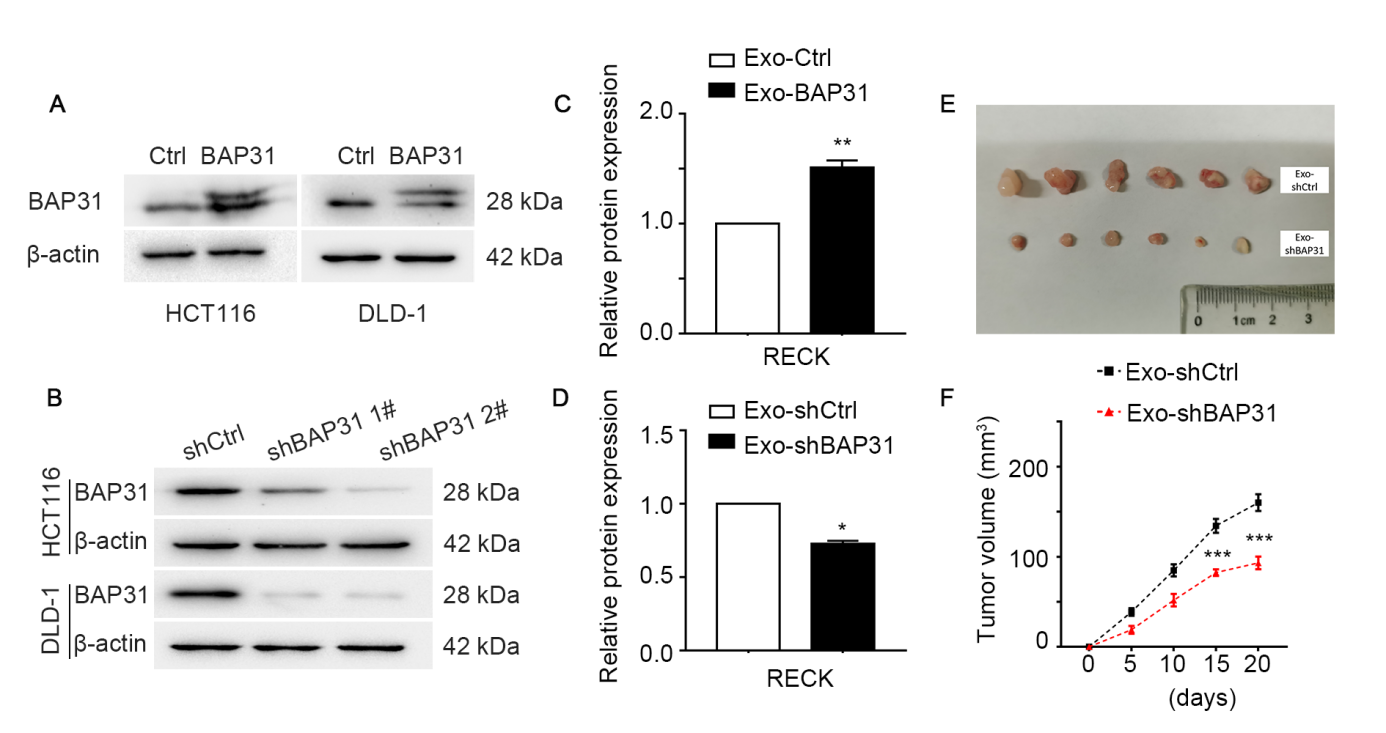
Supplementary Figure2. Exosomes from BAP31-Overexpressing CRCs Promoted Fibroblast Activation.** **(A)** Western blot analysis shows the BAP31 expression in CRCs transfected with pcDNA3.1(-) with BAP31-Flag. **(B)** Western blot analysis shows the BAP31 expression in CRCs transfected with plko.1-puro-shBAP31. Fibroblasts were treated by exosomes from BAP31-overexpressing/BAP31-knockdown CRCs. **(C, D)** ImageJ software analyzes the relative quantification of the RECK expression in fibroblasts. Mean  ± SEM are provided (n = 3). * *p* < 0.05, ** *p* < 0.01. Mouse fibroblasts (NIH/3T3) were treated by exosomes from BAP31-knockdown CRCs. Mouse CRCs (MC38)were mixed with the fibroblasts, and then injected subcutaneously into BALB/C mice. We sacrificed the mice at days 20 and harvested the tumors. **(E)** Images show the isolated tumors from the experimental mice. **(F)** Image J software analyzes the tumor volume every 5 days (n = 6). *** *p* < 0.001.

**
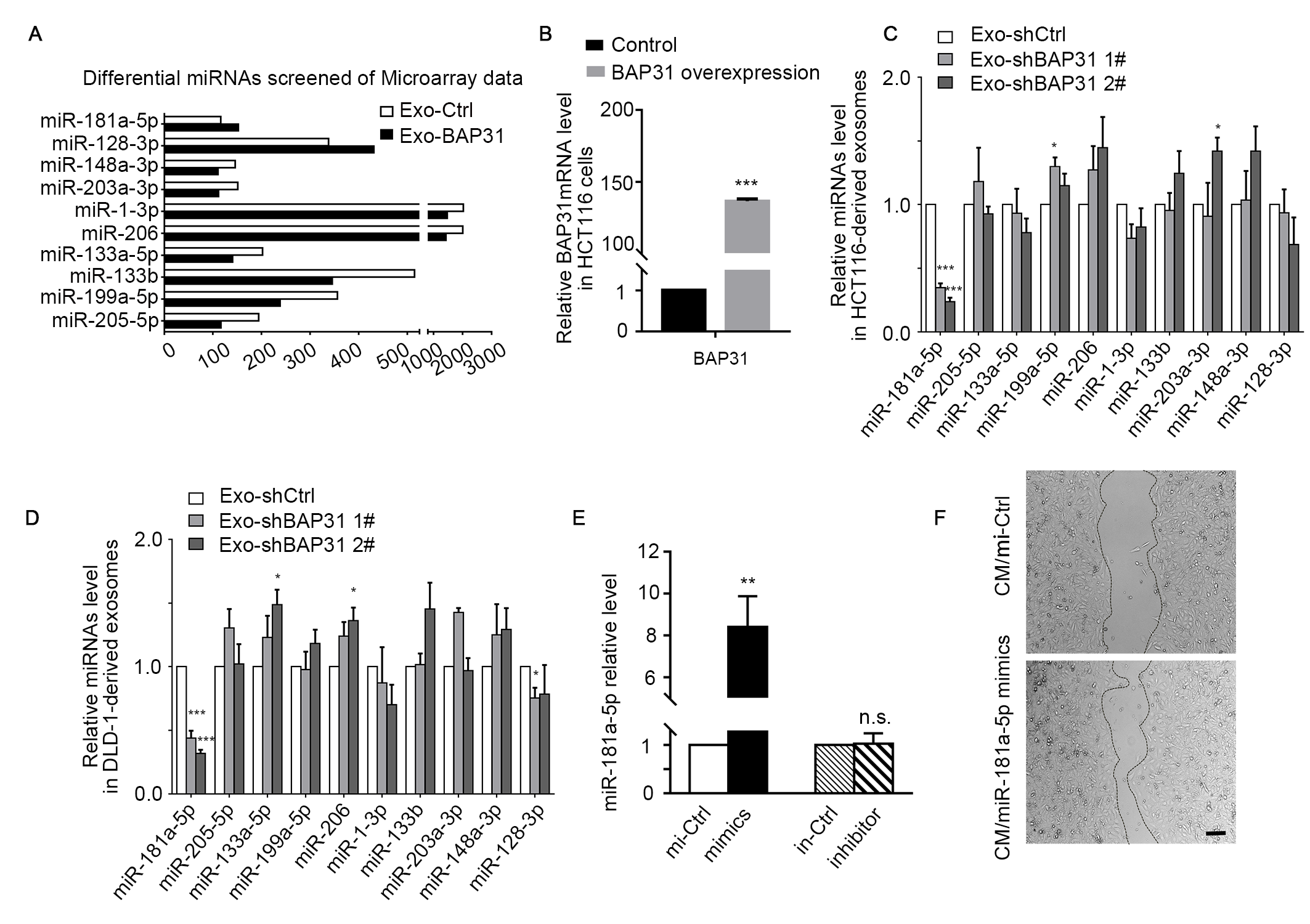
**

**Supplementary Figure3. Exosomal miR-181a-5p was Positively Regulated by the Expression of BAP31.** **(A)** Microarray data is selected to show the difference of exosomal miRNAs from BAP31-overexpressing CRCs. The screening condition was presences ≥ 100 reads, and |log2 (fold change)| ≥ 0.35. **(B)** qPCR analysis shows the BAP31 expression in HCT116 cells. Mean ± SEM are provided (n = 3). *** *p* < 0.001. **(C)** qPCR analysis shows the expression of exosomal miRNAs from BAP31-knockdown HCT116. Mean  ± SEM are provided (n = 3). * *p* < 0.05, *** *p* < 0.001. **(D)** qPCR analysis shows the expression of exosomal miRNAs from BAP31-knockdown DLD-1. Mean  ± SEM are provided (n = 3). * *p* < 0.05, *** *p* < 0.001. **(E)** qPCR analysis shows the miR-181a-5p expression in fibroblasts transfected with miR-181a-5p mimics/inhibitor. Mean  ± SEM are provided (n = 3). ** *p* < 0.01, n.s. non-significance. **(F)** Wound healing assay shows the effect of fibroblasts on endothelial cells migration. Scale bar, 100μm.


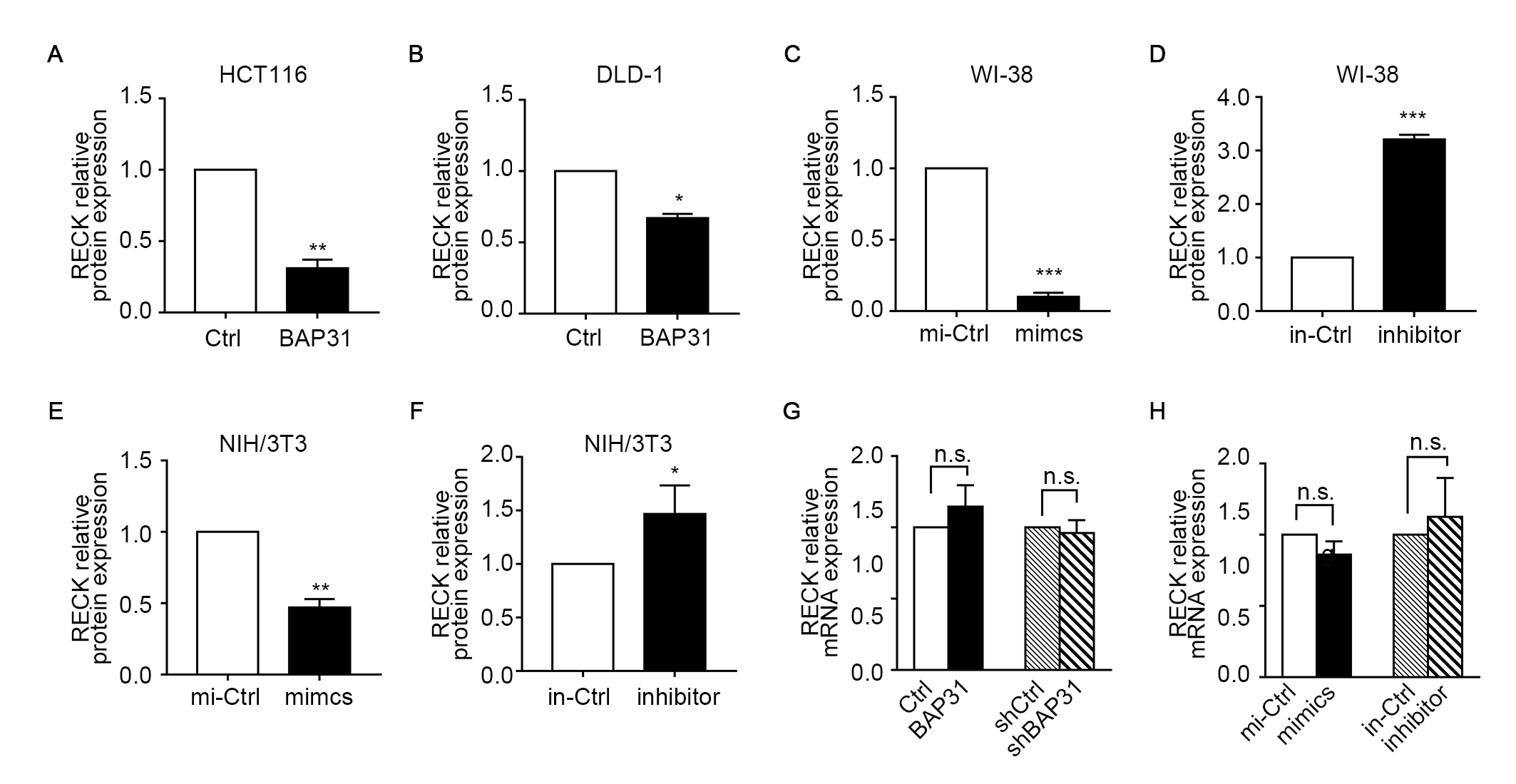


**Supplementary Figure4. The 3’UTR of RECK was a Functional Target of miR-181a-5p.** **(A, B)** Fibroblasts were treated by exosomes from BAP31-overexpressing CRCs. ImageJ software analyzes the relative quantification of the RECK in the fibroblasts. Mean ± SEM are provided (n = 3). * *p* < 0.05, ** *p* < 0.01. Fibroblasts were transfected with miR-181a-5p mimics/inhibitor. **(C, D)** ImageJ software analyzes the relative quantification of the RECK in WI-38. Mean  ± SEM are provided (n = 3). *** *p* < 0.001. **(E, F)** ImageJ software analyzes the relative quantification of the RECK in NIH/3T3. Mean  ± SEM are provided (n = 3). * *p* < 0.05, ** *p* < 0.01. **(G)** ImageJ software analyzes the relative quantification of the RECK in fibroblasts coculturated with exosomes from BAP31-overexpressing/BAP31-knockdown CRCs. Mean ± SEM are provided (n = 3). n.s. non-significance. **(H)** ImageJ software analyzes the relative quantification of the RECK in fibroblasts transfected with miR-181a-5p mimics/inhibitor. Mean ± SEM are provided (n = 3). n.s. non-significance.

**
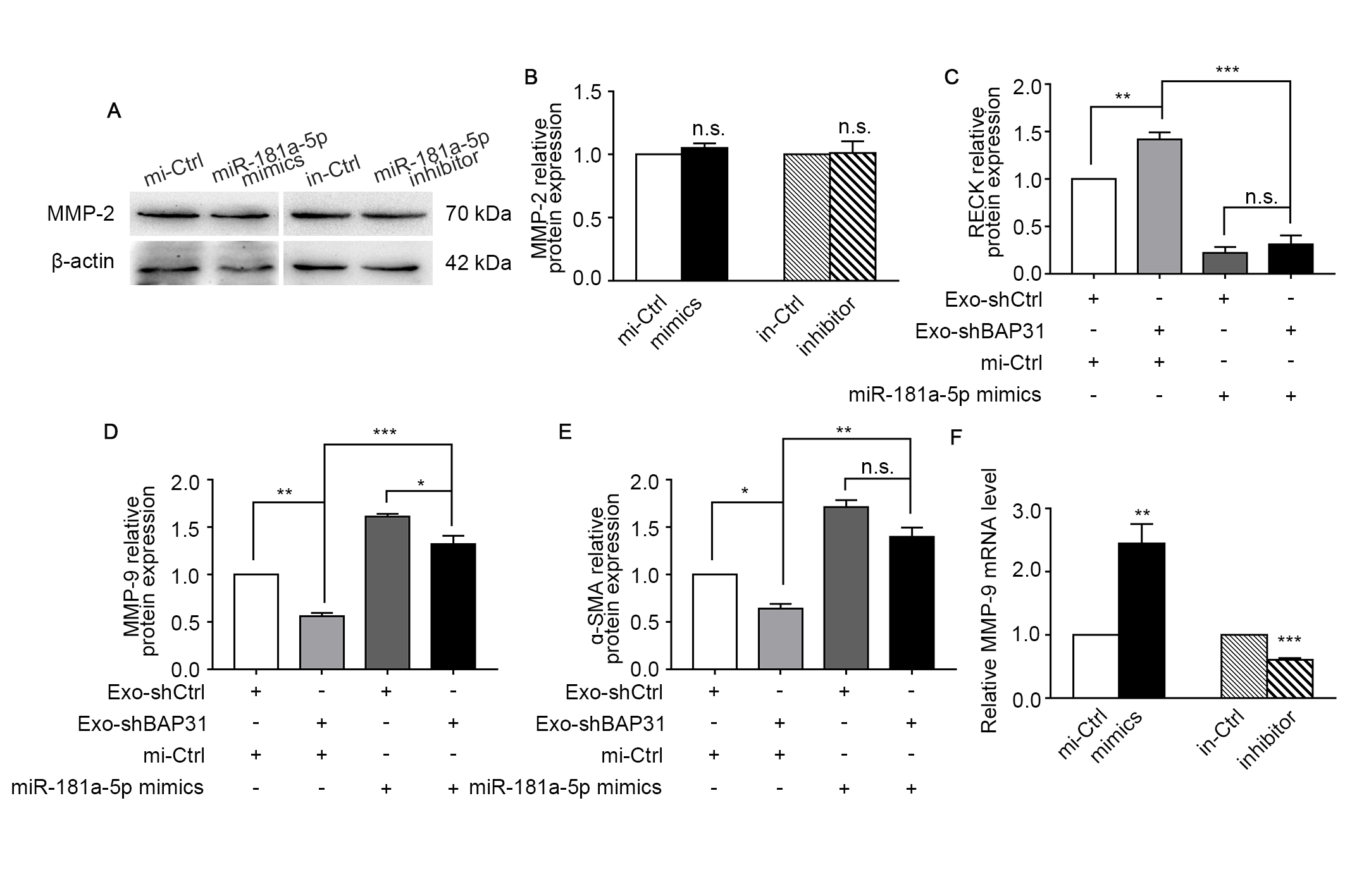
**

**Supplementary Figure5. miR-181a-5p Activated Fibroblasts by by Upregulating MMP-9 and Phosphorylation of smad2/3.** Fibroblasts were transfected with miR-181a-5p mimics/inhibitor. **(A)** Western blot analysis shows the MMP-2 expression in the fibroblasts. **(B)** ImageJ software analyzes the relative quantification of the MMP-2 in the fibroblasts. Mean ± SEM are provided (n = 3). n.s. non-significance. Fibroblasts were transfected with miR-181a-5p mimics or treated by exosomes from BAP31-knockdown CRCs. **(C, D, E)** ImageJ software analyzes the relative quantification of RECK, MMP-9 and α-SMA in the fibroblasts. Mean ± SEM are provided (n = 3). * *p* < 0.05, ** *p* < 0.01, *** *p* < 0.001, n.s. non-significance. **(F)** ImageJ software analyzes the relative quantification of the MMP-9 mRNA in fibroblasts transfected with miR-181a-5p mimic/inhibitor. Mean ± SEM are provided (n = 3). ** *p* < 0.01, *** *p* < 0.001.
