# Supplementary material for: The BAP31/miR-181a-5p/RECK axis promotes angiogenesis in colorectal cancer via fibroblast activation
Source: Front Oncol. 2023 Feb 21;13:1056903. doi: 10.3389/fonc.2023.1056903 (PMC9989165; doi:10.3389/fonc.2023.1056903)
Supplement: Supplementary file 2 [file Table_1.docx]

1. **Supplementary Table1.** List of antibodies

| Names | Manufacturer |
| --- | --- |
| **Primary antibodies** |  |
| β-actin Mouse mAb | CST |
| GAPDH Rabbit mAb | CST |
| BAP31 Rabbit mAb | Sigma |
| Alix Rabbit mAb | Wanlei |
| CD63 Rabbit mAb | Wanlei |
| TSG101 Rabbit mAb | Wanlei |
| GM130 Rabbit mAb | Wanlei |
| CD31 Rabbit mAb | Abcam |
| ɑ-SMA Rabbit mAb | Wanlei |
| RECK Rabbit mAb | Boster |
| MMP-9 Rabbit mAb | proteintech |
| **Blocking antibodies** |  |
| Anti-rabbit IgG Fab2(Flor) 555 | CST |
| Anti-mouse IgG Fab2(Flor) 555 | CST |
