# Supplementary material for: The BAP31/miR-181a-5p/RECK axis promotes angiogenesis in colorectal cancer via fibroblast activation
Source: Front Oncol. 2023 Feb 21;13:1056903. doi: 10.3389/fonc.2023.1056903 (PMC9989165; doi:10.3389/fonc.2023.1056903)
Supplement: Supplementary file 3 [file Table_2.docx]

**Supplementary Table2.** Primer sequences for qPCR

| Primer name | primer sequence (5’→3’) |
| --- | --- |
| **mRNA Primers** |  |
| GAPDH-Forward | GACAGTCAGCCGCATCTTCT |
| GAPDH-Reverse | TTAAAAGCAGCCCTGGTGAC |
| RECK-Forward | AGCAACCGAGCCCGTATGT |
| RECK-Reverse | CCGAGTAGGCAGCACACACA |
| MMP9-Forward | TTGACAGCGACAAGAAGTGG |
| MMP9-Reverse | ACATAGGGTACATGAGCGCC |
| **miRNA Primers** |  |
| U6-Forward | CTCGCTTCGGCAGCACA |
| U6-Reverse | AACGCTTCACGAATTTGCGT |
| miRNAs-Reverse | CTCAACTGGTGTCGTGGA |
| cDNA-miR-181a-5p | CTCAACTGGTGTCGTGGAGTCGGCAATTCAGTTGAGACTCACC |
| miR-181a-5p-Forward | CGCTGTCGGTGAGTCTCA |
| cDNA-miR-128-3p | CTCAACTGGTGTCGTGGAGTCGGCAATTCAGTTGAGAAAGAGA |
| miR-128-3p-Forward | ACAGTGAACCGGTCTCTTTCT |
| cDNA-miR-148a-3p | CTCAACTGGTGTCGTGGAGTCGGCAATTCAGTTGAGACAAAGT |
| miR-148a-3p-Forward | gTCAGTGCACTACAGAACTTTG |
| cDNA-miR-203a-3p | CTCAACTGGTGTCGTGGAGTCGGCAATTCAGTTGAGCTAGTGG |
| miR-203a-3p-Forward | ggGTGAAATGTTTAGGACCACT |
| cDNA-miR-1-3p | CTCAACTGGTGTCGTGGAGTCGGCAATTCAGTTGAGATACATAC |
| miR-1-3p-Forward | gcgcTGGAATGTAAAGAAGTATG |
| cDNA-miR-206 | CTCAACTGGTGTCGTGGAGTCGGCAATTCAGTTGAGCCACACA |
| miR-206-Forward | GAATGTAAGGAAGTGTGTGGCT |
| cDNA-miR-133a-5p | CTCAACTGGTGTCGTGGAGTCGGCAATTCAGTTGAGATTTGGT |
| miR-133a-5p-Forward | ggggAGCTGGTAAAATGGAAC |
| cDNA-miR-133b | CTCAACTGGTGTCGTGGAGTCGGCAATTCAGTTGAGTAGCTGG |
| miR-133b-Forward | GTCCCCTTCAACCAGCTACT |
| cDNA-miR-199a-5p | CTCAACTGGTGTCGTGGAGTCGGCAATTCAGTTGAGGAACAGG |
| miR-199a-5p-Forward | cCCCAGTGTTCAGACTACCT |
| cDNA-miR-205-5p | CTCAACTGGTGTCGTGGAGTCGGCAATTCAGTTGAGCAGACT |
| miR-205-5p-Forward | CCTTCATTCCACCGGAGTC |
