# Supplementary material for: The BAP31/miR-181a-5p/RECK axis promotes angiogenesis in colorectal cancer via fibroblast activation
Source: Front Oncol. 2023 Feb 21;13:1056903. doi: 10.3389/fonc.2023.1056903 (PMC9989165; doi:10.3389/fonc.2023.1056903)
Supplement: Supplementary file 4 [file Table_3.docx]

**Supplementary Table3.** Sequences of synthetic miRNA mimics and inhibitor.

| Primer name | Primer sequence (5’→3’) |
| --- | --- |
| mi-Ctrl-sence | UUCUCCGAACGUGUCACGUTT |
| mi-Ctrl anti-sence | ACGUGACACGUUCGGAGAATT |
| miR-181a-5p mimics-sence | AACAUUCAACGCUGUCGGUGAGU |
| miR-181a-5p mimics anti-sence | UCACCGACAGCGUUGAAUGUUUU |
| in-Ctrl | CAGUACUUUUGUGUAGUACAA |
| miR-181a-5p inhibitor | ACUCACCGACAGCGUUGAAUGUU |
