# Supplementary material for: The BAP31/miR-181a-5p/RECK axis promotes angiogenesis in colorectal cancer via fibroblast activation
Source: Front Oncol. 2023 Feb 21;13:1056903. doi: 10.3389/fonc.2023.1056903 (PMC9989165; doi:10.3389/fonc.2023.1056903)
Supplement: Supplementary file 5 [file Table_4.docx]

**Supplementary Table4.** Target-predictions of miR-181a-5p

|  | TargetScan | | miRDB | miRanda | |
| --- | --- | --- | --- | --- | --- |
|  | Total context++ score | Aggregate PCT | Target Score | mirSVR score | PhastCons score |
| ADAMTS5 | -0.43 | 0.84 | 84 | -0.177 | 0.6255 |
| DEPTOR | -0.38 | 0.69 | 82 | -0.2438 | 0.5569 |
| ARNT2 | -0.2 | 0.78 | 78 | -0.7032 | 0.8119 |
| WIF1 | -0.18 | 0.44 | 61 | -1.1692 | 0.7414 |
| RECK | -0.11 | 0.46 | 83 | -1.0873 | 0.7403 |
| HIPK1 | -0.09 | 0.41 | 70 | -0.8349 | 0.7813 |
| NAA15 | -0.03 | < 0.1 | 54 | -0.8582 | 0.6123 |
